# Ferritin and procalcitonin serve as discriminative inflammatory biomarkers and can predict the prognosis of severe fever with thrombocytopenia syndrome in its early stages

**DOI:** 10.3389/fmicb.2023.1168381

**Published:** 2023-04-18

**Authors:** Keping Chen, Huidi Sun, Yu Geng, Chuankun Yang, Chun Shan, Yuxin Chen

**Affiliations:** ^1^Clinical Laboratory, Zhongda Hospital, Medical School, Southeast University, Nanjing, China; ^2^Department of Infectious Diseases, Nanjing Drum Tower Hospital Clinical College of Nanjing University of Chinese Medicine, Nanjing, Jiangsu, China; ^3^Department of Infectious Diseases, Zhongda Hospital, Southeast University, Nanjing, China; ^4^Clinical Laboratory, Nanjing Drum Tower Hospital, Nanjing University, Nanjing, China

**Keywords:** severe fever with thrombocytopenia syndrome, bunyavirus, inflammatory biomarker, ferritin, procalcitonin, C-reactive protein, white blood cells, prognosis

## Abstract

**Introduction:**

Severe fever with thrombocytopenia syndrome (SFTS) is an emerging infectious disease with high mortality. The pathophysiology of SFTS remains unclear. Hence, the identification of inflammatory biomarkers for SFTS is crucial for the timely management and prevention of disease severity.

**Methods:**

A total of 256 patients with SFTS were divided into a survivor group and a non-survivor group. Classical inflammatory biomarkers such as ferritin, procalcitonin (PCT), C-reactive protein (CRP), and white blood cells were investigated for their association with viral load and the clinical significance for predicting the mortality of patients with SFTS.

**Results:**

Serum ferritin and PCT showed a positive association with viral load. Ferritin and PCT levels in non-survivors were significantly higher than those in survivors at 7–9 days from symptom onset. The area under the receiver operating characteristic curve (AUC) values of ferritin and PCT for predicting the fatal outcome of SFTS were 0.9057 and 0.8058, respectively. However, the CRP levels and WBC counts exhibited a weak association with viral load. The AUC value of CRP for predicting mortality was more than 0.7 at 13–15 days from symptom onset.

**Discussion:**

Ferritin and PCT levels, especially ferritin, could be potential inflammatory biomarkers for predicting the prognosis of patients with SFTS in its early stages.

## 1. Introduction

Severe fever with thrombocytopenia syndrome (SFTS) is an emerging infectious disease caused by a novel bunyavirus (SFTS virus, SFTSV) (Seo et al., 2021). The major clinical characteristics of SFTS include high fever, thrombocytopenia, hemorrhage, and multiple organ failure in severe cases (Kwon et al., 2021). Since its first description in China in 2011, SFTS has been reported in Japan, South Korea, Taiwan, and Vietnam, and the number of patients with SFTS has increased each year in the eastern countries and regions (Yu et al., 2011; Tran et al., 2019; Lin et al., 2020). However, due to the lack of effective treatment options, patients with SFTS are being treated using conservative treatment strategies. Recently, some studies reported that favipiravir treatment could significantly reduce the case fatality rate of SFTS (Li et al., 2021; Suemori et al., 2021; Yuan et al., 2021). However, favipiravir treatment could highly benefit SFTS patients aged ≤70 years (Yuan et al., 2021), or patients with low-viral load (Li et al., 2021; Yuan et al., 2021). Advanced age and high viral load are risk factors for poor progress (Seo et al., 2021; Yoo et al., 2021). These contribute to a high mortality rate of 32.6% for SFTS (Choi et al., 2016; Li et al., 2018). The pathophysiological mechanisms of SFTS have not been completely elucidated, but it has been linked to a high viral load and an inflammatory response (Seo et al., 2021). Hence, early detection and identification of SFTS biomarkers are important measures to prevent the severity of SFTS.

Clinical laboratory parameters and disease biomarkers are necessary for improving the treatment and management of patients with SFTS. Some biomarkers may be used as a clinical reference for assessing severity and predicting prognosis. White blood cells (WBC) and C-reactive protein (CRP) are classical inflammatory markers in bacterial sepsis compared with viral diseases and other inflammatory conditions (Kim et al., 2017; Shin et al., 2018). However, the role of WBC and CRP in the clinical diagnosis and treatment of SFTSV infection remains controversial and unclear (Deng et al., 2013; Sun et al., 2014; Kawaguchi et al., 2020; He and Liu, 2021; Xu et al., 2021). For the clinical diagnosis of bacterial infection, procalcitonin (PCT) is also used as a reliable inflammatory biomarker with high sensitivity and specificity. However, PCT is not significantly increased in viral infections (Lippi and Sanchis-Gomar, 2017). Recently, ferritin was confirmed as an inflammation and infection biomarker in the diagnosis of viral and bacterial infections (Franco-Martinez et al., 2021; Zhou et al., 2021). Whether ferritin has a clear advantage as a reliable biomarker for SFTSV infection needs to be further studied. In this study, we aimed to analyze the clinical significance of WBC, CRP, PCT, and, ferritin as inflammatory biomarkers for SFTSV infection, and determine the association between these biomarkers and the prognosis of patients with SFTS. Early identification of risk factors associated with the fatal outcome of SFTS would be beneficial for timely treatment and management.

## 2. Materials and methods

### 2.1. Patients and clinical samples

We collected the demographical and clinical characteristics of SFTS patients who were admitted to the Zhongda Hospital and Nanjing Drum Tower Hospital from 2021 to 2022. The patients were confirmed for SFTSV infection by detecting the SFTSV RNA with real-time reverse transcription polymerase chain reaction (RT-PCR). The patients were divided into the survivor and non-survivor groups based on the outcome of patients with SFTS. The serum samples were obtained from each patient during the hospitalization period and stored at −80°C until further analysis. The study protocol was approved by the Ethics Committee of Zhongda Hospital and Nanjing Drum Tower Hospital.

### 2.2. Quantification of viral RNA load

Total viral RNA was extracted from the serum samples using an automatic nucleic acid extraction and purification system (NP968-C) with an extraction and purification kit (TIANLONG, Xi’an, China). SFTSV RNA load in serum samples was measured using the SFTSV quantification kit (DAAN GENE, Guangzhou, China), according to the manufacturer’s instructions, and the RT-PCR was performed in the ABI7500 system (Applied Biosystems, Foster City, CA, United States). The human actin gene was used as an internal control.

### 2.3. Measurement of WBC, serum CRP, ferritin, and procalcitonin

Serum ferritin was measured using an enzyme chemiluminescence immunoassay. In brief, an anti-ferritin antibody labeled with alkaline phosphatase, and magnetic beads coated with a second anti-ferritin antibody, were combined with serum, which forms antibody-ferritin-antibody “sandwich” complexes. Then, the chemiluminescence substrate Lumi-Phos530 was added, and the luminescence produced in the complexes was measured with a Beckman Coulter DxI 800 Access (Beckman Coulter, Brea, CA, United States).

Serum PCT was measured using an electrochemiluminescence immunoassay. In brief, serum samples, biotinylated monoclonal antibody, and [Ru(bpy)_3_]^2+^labeled monoclonal antibody were incubated together to form an antibody–antigen–antibody “sandwich” complex. Then magnetic beads coated with streptomycin were added for incubation. The complex and magnetic beads were bound by the actions of biotin and streptavidin After adsorption and washing, a certain voltage was applied to the electrode to make the complex chemiluminescent, and the luminescence intensity was measured with Roche cobas 8,000 (Roche Diagnostics, Mannheim, Germany).

For CRP measurement, polystyrene particles coated with anti-CRP monoclonal antibody and the samples were incubated together to form a complex that can make the light beam scatter. CRP levels were quantified by measuring the intensity of the scattered light with the BN ProSpec System (Siemens Healthcare, Marburg, Germany). WBC was counted by the SYSMEX XN automatic hematology analyzer and matched reagents (SYSMEX, Shanghai, China).

### 2.4. Statistical analysis

Continuous variables were presented as mean ± standard deviations (SD) and analyzed using the Mann–Whitney *U* test. Categorical variables were represented as numbers and percentages and analyzed using Pearson’s chi-square test or Fisher’s exact test. Pearson’s correlation tests were used to measure the association between inflammatory biomarkers and viral load. All tests of significance were two-tailed, and value of ps less than 0.05 were considered statistically significant. Statistical analysis was performed using SPSS 18.0 and GraphPad Prism 8.0.2 software.

## 3. Results

### 3.1. Clinical characteristics of the patients

A total of 256 patients with SFTS were included in this study, consisting of 197 (76.95%) survivors and 59 (23.05%) non-survivors. Detailed clinical characteristics of these patients were shown in Table 1. In our dataset, the non-survivors were older than the survivors. In addition, the duration of hospitalization and clinical course in the non-survivor group were significantly shorter than those in the survivor group (*p* < 0.001). In the non-survivor group, conditions such as respiratory dysfunction, central nervous system disorder, multiple-organ failure, shock, and sepsis were more common compared to those in the survived group (*p* < 0.001). These complications were the risk factors for the fatal outcome of patients with SFTS. However, we observed that hypertension, coronary artery disease, and diabetes were not significantly different between the survivor and non-survivor groups.

**Table 1:** Demographic characteristics of the patients with SFTS.

Variables	Survivors (*n* = 197)	Non-survivors (*n* = 59)	*p* value
Male, *n* (%)	102 (51.78%)	30 (50.85%)	*p* = 0.900
Age (years)	58.96 ± 12.80	66.24 ± 9.54	*p* < 0.001
Time from symptom onset to hospitalization (days)	8.54 ± 3.97	7.81 ± 2.63	*p* = 0.226
Duration of hospitalization	14.32 ± 8.59	7.07 ± 6.18	*p* < 0.001
Duration of clinical course	21.86 ± 9.10	13.88 ± 6.33	*p* < 0.001
Comorbidity *n* (%)			
Hypertension	51 (25.89%)	19 (32.20%)	*p* = 0.340
Coronary artery disease	5 (2.54%)	2 (3.39%)	*p* = 0.725
Diabetes	22 (11.17%)	5 (8.47%)	*p* = 0.555
Complication *n* (%)			
Respiratory failure	5 (2.54%)	16 (27.12%)	*p* < 0.001
Central nervous syndrome	15 (7.61%)	15 (25.42%)	*p* < 0.001
Multiple-organ failure	56 (28.43%)	39 (66.10%)	*p* < 0.001
Shock	4 (2.03%)	15 (25.89%)	*p* < 0.001
Sepsis	9 (4.57%)	14 (23.72%)	*p* < 0.001
Hemorrhage	2 (1.02%)	2 (3.39%)	*p* = 0.197

### 3.2. Inflammatory biomarkers levels associated with viral load

**Figure 1:**
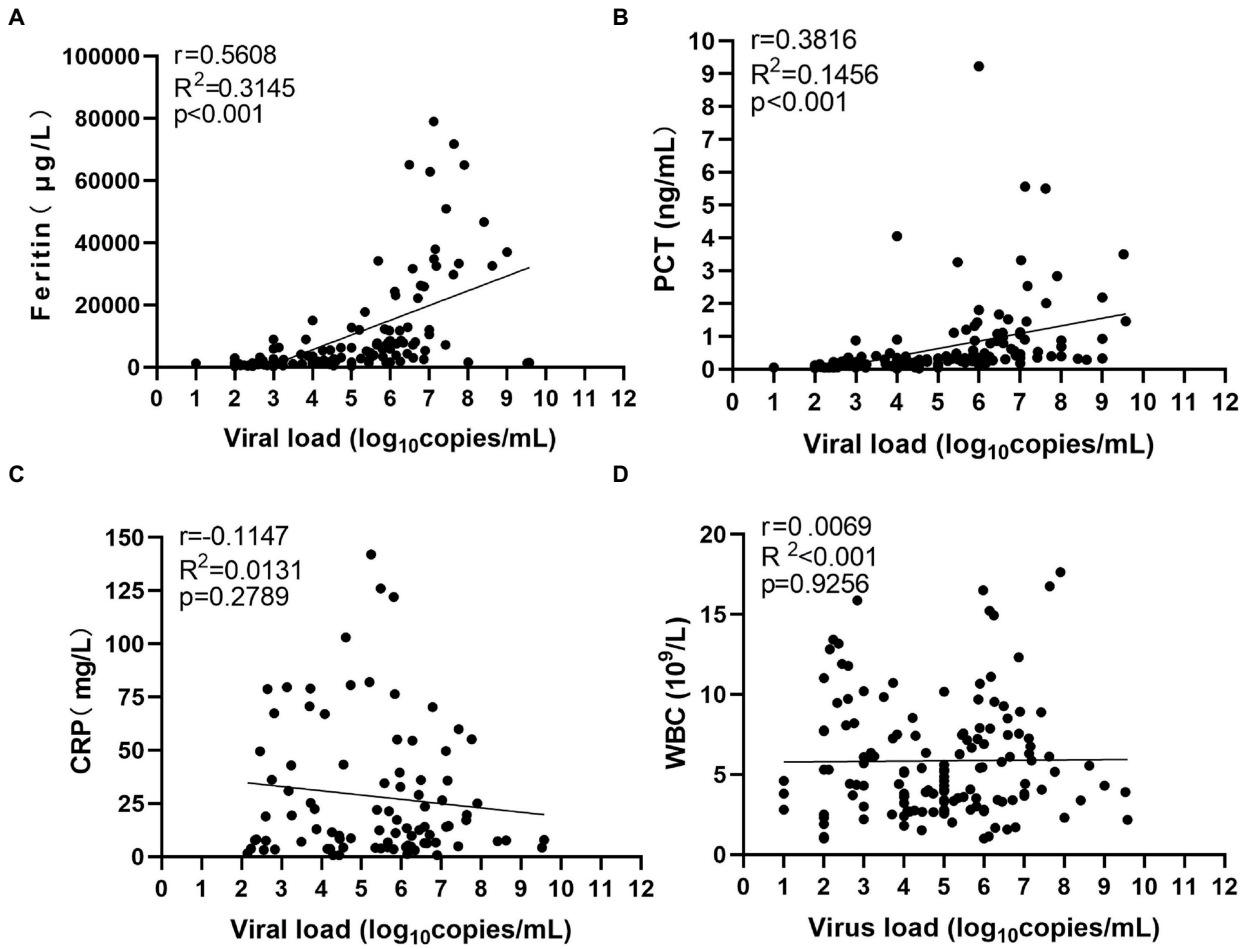
Correlation between serum viral load and inflammatory biomarkers. R^2^ represents the coefficient of the determination, and r represents the Pearson correlation coefficient, with r values of 0–0.3, 0.3–0.5, and >0.5 indicating weak, moderate, and strong correlation, respectively. *p* values less than 0.05 were considered statistically significant.

To find potential biomarkers for SFTS, we first investigated the association of serum viral load with inflammatory markers. The viral load and levels of inflammatory biomarkers were quantified during the hospitalization period. Our results show that serum ferritin showed the strongest positive association with viral load (Figure 1A). Furthermore, serum PCT levels exhibited a moderate association with viral load (Figure 1B). However, the serum CRP levels and WBC counts exhibited a weak association with viral load (Figures 1C,D). Together, these results show that ferritin is a potential marker for inflammation in patients with SFTS, and can indicate the level of viral replication and the progression of SFTS compared to serum CRP and WBC counts.

### 3.3. Kinetics of inflammatory biomarker levels in survivors and non-survivors

**Figure 2:**
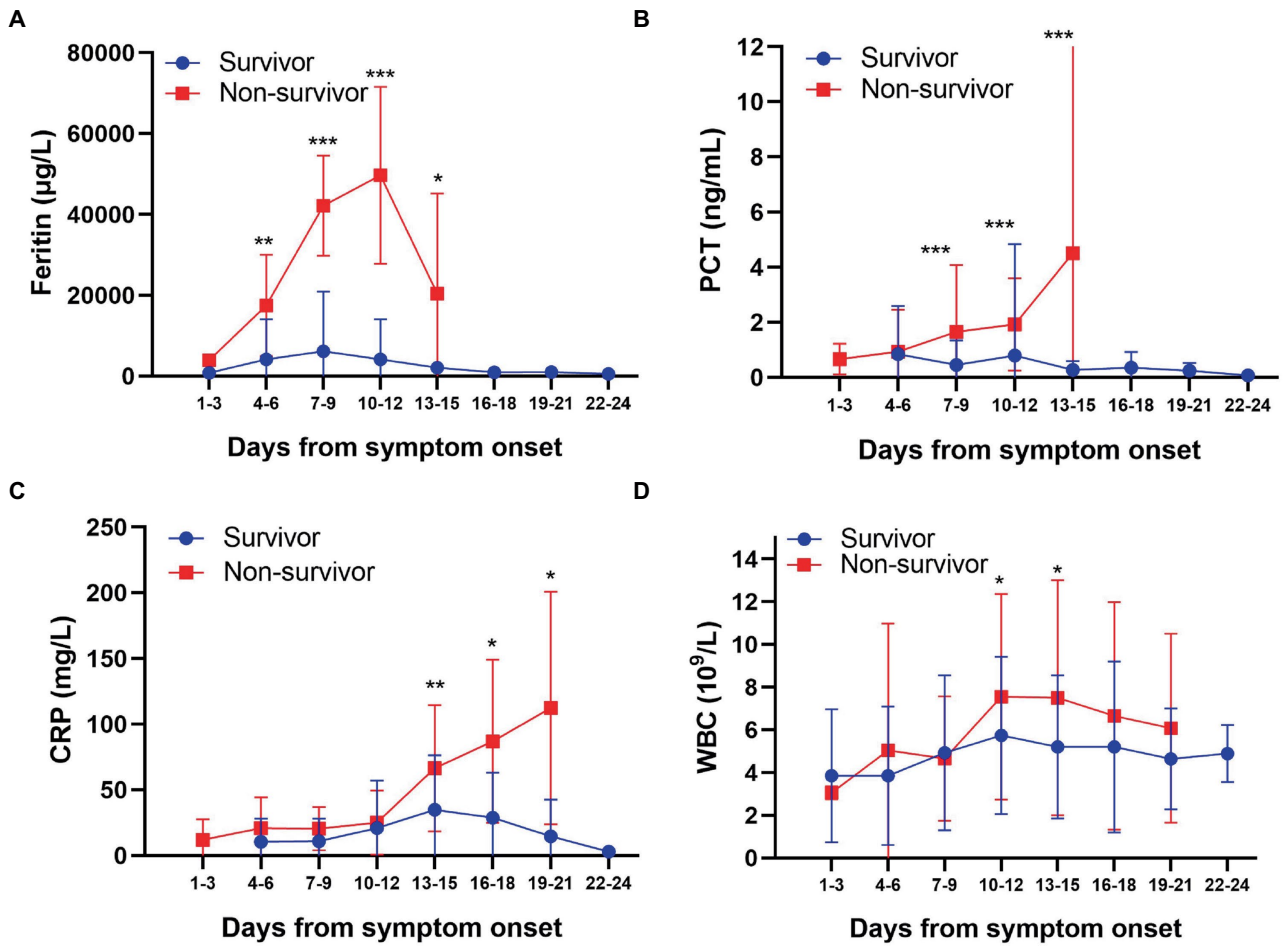
Kinetic comparison of inflammatory biomarkers between survivors and non-survivors. *p < 0.05; **p < 0.01; ***p < 0.001. Blue, survivors; red, non-survivors.

We then looked at the kinetics of the change in inflammatory biomarkers among the patients (Figure 2). We observed that the serum ferritin levels in all the SFTS patients were significantly higher than the upper limit of the reference range (200 μg/L) during the hospitalization, especially in non-survivors. The highest level of serum ferritin measured was 38,860 μg/L in a non-survived subject, which was 194 times more than the upper limit of the reference range. Ferritin levels in non-survivors began to rise steadily from the onset of symptoms and peaked around 10–12 days later. The non-survivors exhibited significantly higher ferritin levels compared to the survivors throughout the clinical course, starting 7–9 days from symptom onset (Figure 2A). Especially, ferritin levels in non-survivors were 12 times higher than those in survivors at 10–12 days from symptom onset.

PCT levels in non-survivors were higher than the upper limit of the reference range (0.5 ng/mL) during the whole clinical course until the patients died. The highest level of serum PCT was 20.31 ng/mL in one non-survived case, which was 40 times more than the upper limit of the reference range. PCT continuously increased from the symptom onset to 13–15 days from symptom onset in non-survivors. However, PCT in most survivors was within the reference range (<0.5 ng/mL) during the hospitalization period. These results show that the serum PCT levels in non-survivors were significantly higher than those in survivors from 7–9 days after symptom onset (Figure 2B).

Furthermore, CRP levels in all the SFTS patients were far higher than the upper limit of the reference range (10 mg/L) during the majority of the hospitalization period, especially in non-survivors. The CRP levels in non-survivors continuously increased during the clinical course until the patient died. However, the CRP levels in non-survivors were not significantly higher than those in survivors at 7–9 days from symptom onset, which was different compared to the serum ferritin and PCT profiles. The CRP levels in survivors peaked at 13–15 days from symptom onset and then decreased gradually. The CRP levels in non-survivors were significantly higher than those in survivors from 13–15 days after onset (Figure 2C). Hence, in the later period of SFTS, continuously increased CRP levels were associated with poor outcomes. WBC counts in SFTS patients were generally within the reference range (4-10^9^/L) irrespective of survival, except that WBC counts were below the lower limit of the reference range at 1–3 days from symptom onset (Figure 2D). These results indicate that there was no significant change in WBC counts during the course of SFTS.

Based on the above analysis, we believe that serum ferritin and PCT were appropriate inflammation and infection biomarkers and are associated with fatal outcomes in SFTS patients. Collectively, our results suggested that high ferritin and PCT levels in the early stages of infection and the continuous increase of CRP levels in the later period indicate a poor prognosis in SFTS patients.

### 3.4. Serum ferritin, PCT, and CRP levels predict mortality In SFTS patients

**Figure 3:**
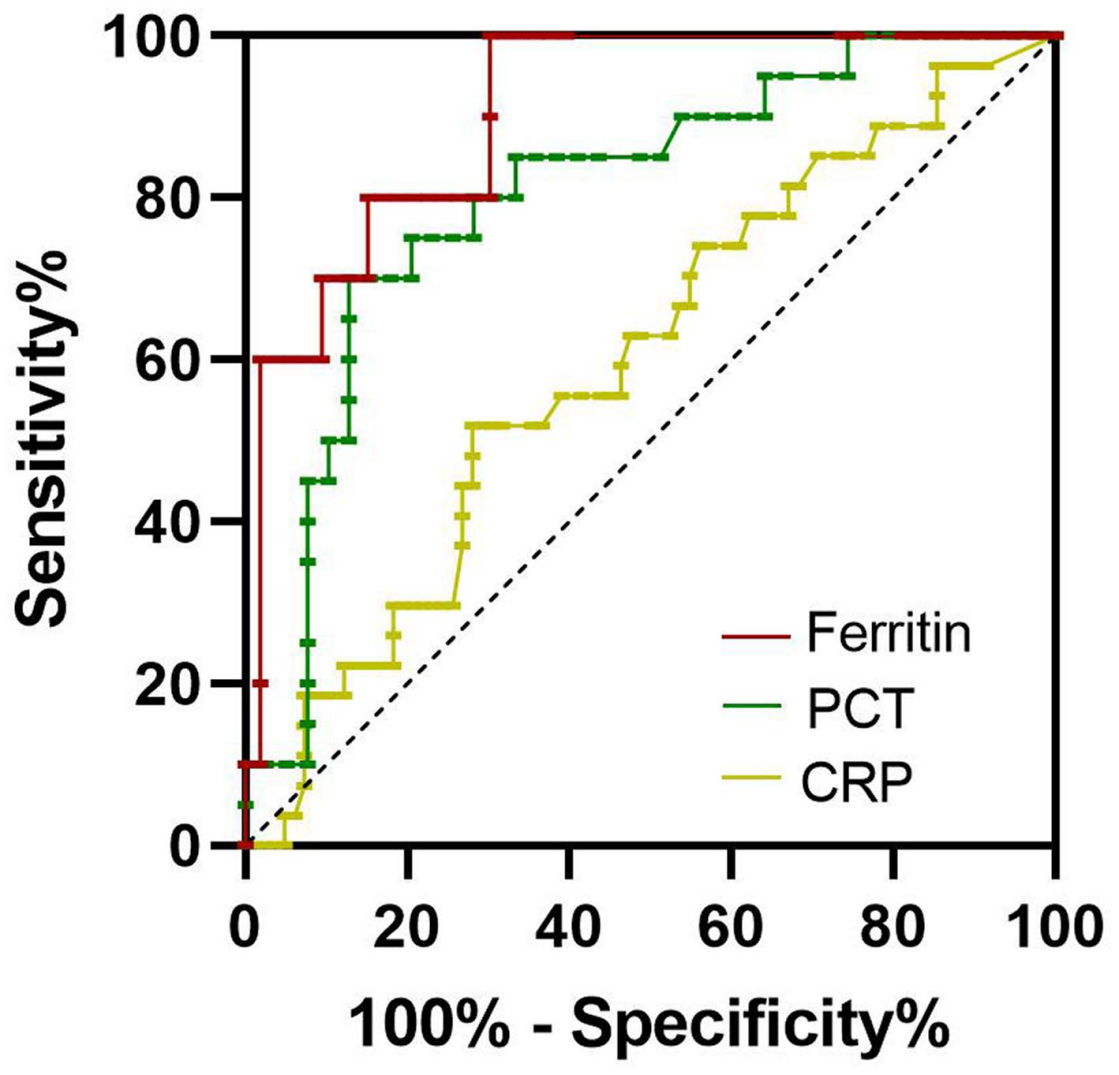
Receiver operating characteristics curves showing the performance of ferritin, PCT, and CRP to predict the mortality of SFTS patients at 7–9 days from symptom onset. The AUC of serum ferritin = 0.9057, the AUC of serum PCT = 0.8058, the AUC of CRP = 0.5996.

**Figure 4:**
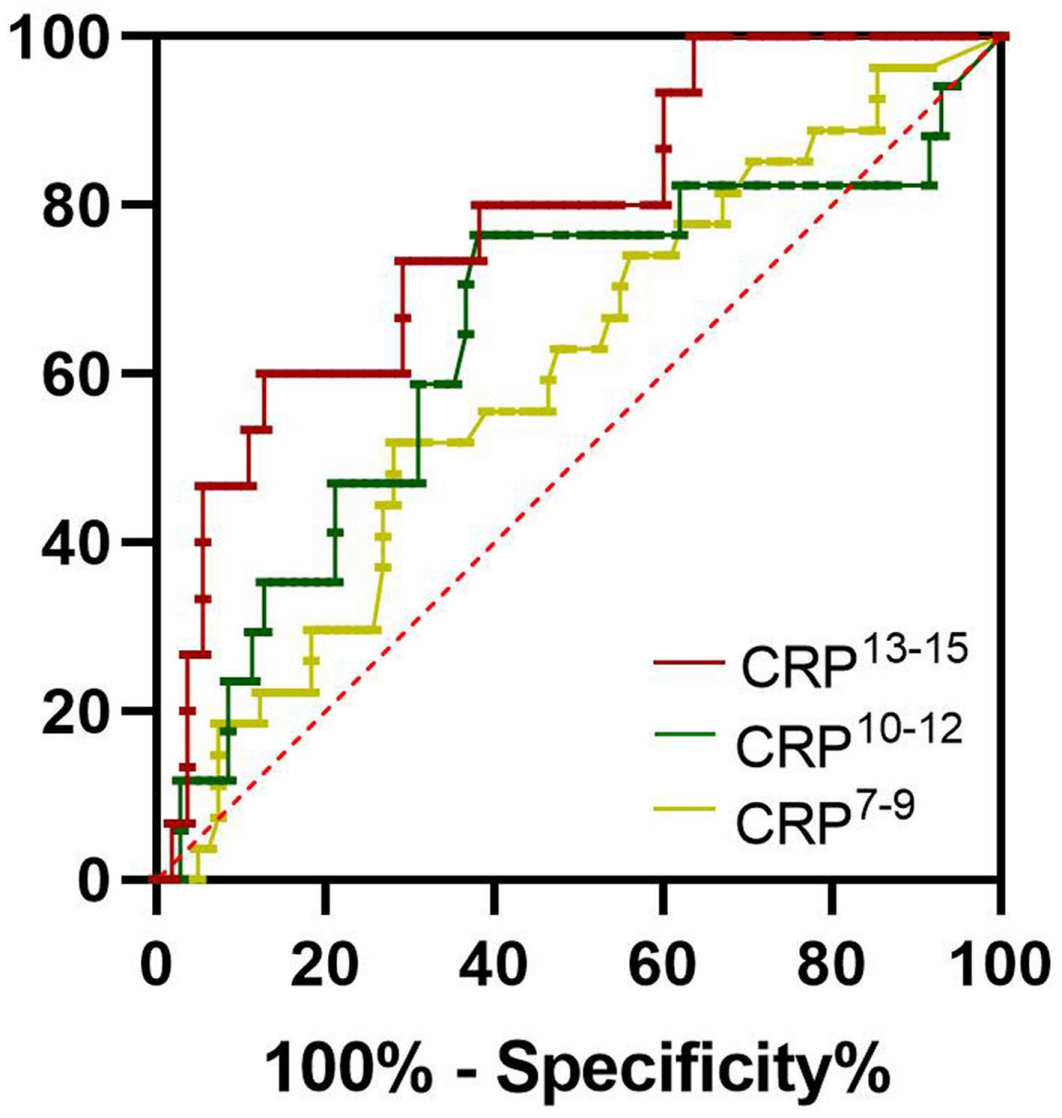
Receiver operating characteristics curves showing the performance of CRP to predict the mortality of SFTS patients at different periods. CRP^7-9^, CRP^10-12^, and CRP^10-12^ indicated the CRP levels at 7–9 days, at 10–12 days, and 13–15 days from symptom onset, respectively. The AUC of CRP^7-9^ = 0.5996, The AUC of CRP^10-12^ = 0.6446, The AUC of CRP^13-15^ = 0.7782.

The levels of serum ferritin, PCT, and CRP in non-survivors were higher than those in survivors. So, in the present study, we evaluated the clinical significance of these inflammatory biomarkers for predicting the outcome of SFTS. The area under the receiver operating characteristic curve (AUC) value of serum ferritin for predicting the mortality of SFTS was 0.9057 (95% CI 0.8201–0.9913, *p* < 0.001) at 7–9 days from symptom onset (Figure 3; Table 2). The optimal cutoff value for serum ferritin was 3,018 μg/L. The AUC value of PCT for predicting the mortality of SFTS was 0.8058 (95% CI 0.6873–0.9243, *p* < 0.001) at 7–9 days from symptom onset. The optimal cutoff value for PCT was 0.3456 ng/mL (Figure 3; Table 2). Based on these results, values greater than 3,018 μg/L for serum ferritin or 0.34 ng/ml for PCT at 7–9 days from symptom onset indicate a higher probability of mortality. However, the AUC value of CRP for predicting the mortality of SFTS was less than 0.7 at 7–9 days and 10–12 days from symptom onset. However, the AUC value of CRP for predicting mortality was more than 0.7 at 13–15 days from symptom onset (Figure 4; Table 2). Hence, serum ferritin and PCT, especially ferritin, were the best inflammatory biomarkers and could predict the probability of mortality at the early stage of SFTS.

**Table 2:** Predictive values of inflammatory biomarkers for the mortality of SFTS.

Biomarkers	AUC	95% CI	value of *p*	Cut-off value	Sensitivity %	Specificity %
Ferritin	0.9057	0.8201–0.9913	<0.0001	3018 μg/L	100	70
PCT	0.8058	0.6873–0.9243	0.0001	0.3456 ng/mL	70	87
CRP^7-9^	0.5996	0.4787–0.7204	0.1217	8.4 mg/L	52	72
CRP^10-12^	0.6446	0.4859–0.8032	0.0651	6.9 mg/L	77	68
CRP^13-15^	0.7782	0.6482–0.9081	0.0010	49.35 mg/L	60	87

## 4. Discussion

CRP is a nonspecific acute-phase inflammatory protein that is produced by hepatocytes and controlled by cytokines such as interleukin-6 (IL-6) and IL-1. Therefore, it is puzzling that CRP levels were generally normal despite the production of these cytokines in SFTS patients (Deng et al., 2013; Sun et al., 2014; Kawaguchi et al., 2020). He et al. found that the CRP levels in SFTS patients were significantly higher than those in the control group (He and Liu, 2021). It has been shown previously that the nonstructural protein of SFTSV can activate the tumor progression locus 2, which promotes the production of IL-10 to suppress the production of IL-6 (Choi et al., 2019). Thus, the low levels of CRP in SFTS patients might be partially explained by the high levels of IL-10. In general, a mild viral infection usually results in either mildly increased or unchanged CRP levels, but a severe viral infection could cause extensive tissue damage, resulting in a significant increase in the CRP levels (He et al., 2021). Our results were consistent with the above observations. In the present study, we observed no significant differences in CRP levels between the survivors and non-survivors during the first 1 to 12 days after symptom onset. However, the CRP levels in non-survivors became significantly higher than those in survivors at 13–15 days from symptom onset, and the AUC of CRP was greater than 0.7, indicating the moderate power of CRP as a biomarker for predicting the SFTS outcome. As the disease progresses, some SFTS patients may develop bacterial co-infection, which may advance to sepsis. More than 23% of patients in non-survivors developed sepsis in the present study. SFTS patients with hemophagocytic lymphohistiocytosis (HLH) were common and had a higher risk of fatal outcome (Jung et al., 2019). These complications may contribute to the increased CRP levels. So, CRP had limited value to estimate disease development, and increased CRP levels at the late stages indicated the fatal outcome of SFTS.

As an acute-phase protein, ferritin is an inflammation and infection biomarker in the diagnosis of viral and bacterial infections; however, ferritin levels are not usually high in acute extracellular bacterial sepsis (Singh et al., 2015). Highly elevated serum ferritin has been reported only in several viral infections, including dengue (Soundravally et al., 2015), chikungunya (Betancur et al., 2015), and viral hepatitis (Mao et al., 2015). Markedly elevated ferritins were found in all of the SFTS patients (Kim et al., 2017), which were consistent with our results. Besides, serum ferritin levels showed the strongest positive association with the viral load. A high ferritin level was indicative of a high viral load and elevated ferritin levels may suggest increased viral replication. The serum ferritin levels in non-survivors were significantly higher than those in survivors from the fourth day after onset. Importantly, the AUC value of ferritin for predicting mortality was 0.91 at 7–9 days from symptom onset, indicating the discriminative power of ferritin as a biomarker. Ferritin levels also increase in HLH patients, especially pediatric HLH (Skinne et al., 2019; Griffin et al., 2020). Many SFTS cases are complicated by HLH, which is a risk factor for poor prognosis in SFTS patients (Jung et al., 2019; Yamauchi et al., 2022). Therefore, increased ferritin levels may be associated with the occurrence of HLH in SFTS. Of course, it is necessary to clarify the mechanism of ferritin increasing in SFTS in future studies. Hence, our results showed ferritin was associated with mortality and could predict the prognosis at the early stage of SFTS development.

PCT levels are generally elevated in bacterial infections, and high serum PCT levels were associated with a greater risk of septic shock and death. PCT was not increased or only slightly increased in viral diseases (Lippi and Sanchis-Gomar, 2017). However, some studies reported PCT levels elevated in SFTS patients and were associated with the outcome of the disease. PCT levels in SFTS patients were significantly higher than those in 2019-nCoV patients and the control individuals (He and Liu, 2021). Moreover, PCT is an independent predictor of encephalitis/encephalopathy for SFTS patients, and as a stable risk factor, PCT levels were associated with mortality in SFTS patients (Xu et al., 2021; Wang et al., 2022). In the present study, we found that the PCT levels in non-survivors were higher than those in survivors and associated with the mortality of the SFTS from 7–9 days after onset. The AUC value of PCT in this period was 0.81, indicating the discriminative power of the biomarker. Collectively, we show that PCT is associated with mortality and can predict the prognosis at the early stage of SFTS development.

In summary, ferritin and PCT levels, especially ferritin, had a strong positive association with SFTSV load and can predict the prognosis of patients with SFTS in its early stage. These results provide further insights into the clinical characteristics associated with the prognosis of SFTS, which in turn contribute to the severity assessment and prognosis prediction.

## Data availability statement

The datasets presented in this study are included in the article, and further inquiries can be available from the corresponding author at a reasonable request.

## Ethics statement

The studies involving human participants were reviewed and approved by the Ethics Committee of Zhongda Hospital and Nanjing Drum Tower Hospital. The patients/participants provided their written informed consent to participate in this study.

## Author contributions

KC and YC conceived and designed this study. HS, CS, and YG collected and organized the data. CY conducted the statistical analysis. KC drafted the manuscript. All authors contributed to the article and approved the submitted version.

## Funding

This study was supported by the Medical Science and Technology Development Foundation of Nanjing (YKK17287). The funders had no role in the study design, data collection, analysis, the decision to publish, or the preparation of the manuscript.

## Conflict of interest

The authors declare that the research was conducted in the absence of any commercial or financial relationships that could be construed as a potential conflict of interest.

## Publisher’s note

All claims expressed in this article are solely those of the authors and do not necessarily represent those of their affiliated organizations, or those of the publisher, the editors and the reviewers. Any product that may be evaluated in this article, or claim that may be made by its manufacturer, is not guaranteed or endorsed by the publisher.
